# Simultaneous Quantitative Analysis of Ginsenosides Isolated from the Fruit of *Panax ginseng* C.A. Meyer and Regulation of HO-1 Expression through EGFR Signaling Has Anti-Inflammatory and Osteogenic Induction Effects in HPDL Cells

**DOI:** 10.3390/molecules26072092

**Published:** 2021-04-06

**Authors:** Eun-Nam Kim, Oryon Kaygusuz, Hyun-Su Lee, Gil-Saeng Jeong

**Affiliations:** College of Pharmacy, Keimyung University, 1095 Dalgubeol-daero, Daegu 42601, Korea; enkimpharm@gmail.com (E.-N.K.); oryonkaygusuz@gmail.com (O.K.); hyunsu.lee@kmu.ac.kr (H.-S.L.)

**Keywords:** epidermal growth factor receptor (EGFR), heme oxygenase 1 (HO-1), ginsenosides, periodontitis, *Panax ginseng*

## Abstract

Periodontitis is a set of chronic inflammatory diseases caused by the accumulation of Gram-negative bacteria on teeth, resulting in gingivitis, pocket formation, alveolar bone loss, tissue destruction, and tooth loss. In this study, the contents of ginsenosides isolated from *Panax ginseng* fruit extract were quantitatively analyzed, and the anti-inflammatory effects were evaluated in human periodontal ligament cells. The major ginsenosides, Re, Ra8, and Rf, present in ginseng fruit were simultaneously analyzed by a validated method using high-performance liquid chromatography with a diode-array detector; Re, Ra8, and Rf content per 1 g of *P. ginseng* fruit extract was 1.01 ± 0.03, 0.33 ± 0.01, and 0.55 ± 0.04 mg, respectively. Ginsenosides-Re, -Ra8, and -Rf inhibited the production of pro-inflammatory factors and the expression of important cytokines in periodontitis by inducing the expression of heme oxygenase 1 (HO-1), promoting osteoblast differentiation of periodontal ligament cells, suppressing alveolar bone loss, and promoting the expression of osteoblast-specific genes, such as *alp*, *opn*, and *runx2*. An inhibitory effect of these ginsenosides on periodontitis and alveolar bone loss was observed via the regulation of HO-1 and subsequent epidermal growth factor receptor (EGFR) signaling. Silencing EGFR with EGFR siRNA confirmed that the effect of ginsenosides on HO-1 is mediated by EGFR. In conclusion, this study evaluated the contents of ginsenosides-Re, -Ra8, and -Rf isolated from *P. ginseng* fruit extract. Therefore, these results provide important basic data for future *P. ginseng* fruit component studies and suggest that ginsenosides Re, Ra8, and Rf have potential as future treatment options for periodontitis.

## 1. Introduction

Periodontitis is a multifactorial chronic disease caused by the accumulation of Gram-negative bacteria on teeth [1] that usually occurs in early adulthood but can also start in childhood or adolescence [2]. Some patients with serious disease, such as localized aggressive periodontitis, do not present classical risk factors [3]. It is uncommon for periodontitis to affect all teeth; usually, only certain tooth and tooth surfaces are impacted [4]. The structure of Gram-negative bacteria is different from that of other bacteria and is surrounded by two cell membranes with a thin layer of peptidoglycan bonded between them [5]. These Gram-negative bacteria are involved in several stages such as inflammation and alveolar bone loss due to the fact of periodontitis, tooth loss, and periodontal tissue destruction. The model for induction of periodontitis using *Porphyromonas gingivalis* (PG)-lipopolysaccharide (LPS) belonging to Gram-negative bacteria is known as a representative model in typical experimental periodontitis studies [6].

The human periodontal ligament is a connective tissue consisting of fibroblastic and osteoblastic cells that play a role in supporting teeth with alveolar bone [7]. Mechanical removal of biofilms from teeth is a traditional approach. Traditionally, the treatment of periodontitis has involved the use of various local and systemic antibiotics; however, the development of antibiotic resistance has been reported [8]. Additionally, while delaying treatment can result in serious damage, if detected early, periodontitis has an excellent prognosis. Any inflammatory periodontal disease may need to be controlled to prevent the colonization and spread of bacterial pathogens [9]. Periodontitis is a caused by accumulation of pathogenic bacteria in teeth. Chronic inflammation caused by periodontitis eventually leads to the loss of periodontal tissues, including alveolar ligaments and alveolar bones [10]. Therefore, suppressing alveolar bone loss and inflammation is an important treatment strategy for periodontitis. In this study, the contents of ginsenosides Re, Ra8, and Rf, isolated from ginseng fruit extract, were analyzed using an established assay, and their effects on periodontitis in human periodontal ligament (HPDL) cells stimulated with PG-LPS were evaluated.

Hemeoxygenase-1 (HO-1) catalyzes the rate-limiting step of heme breakdown through the formation of iron, carbon monoxide (CO), and biliverdin, which is then converted to bilirubin by biliverdin reductase. Previous studies have focused on the antioxidant, anti-inflammatory, and cellular protective functions of HO-1 [11,12]. However, recent studies have reported that the induction of HO-1 through activation of nuclear factor-red blood cell 2-related factor 2 (Nrf2) suppresses oxidative stress and can prevent the progression of osteoporosis [13,14]. Epidermal growth factor receptor (EGFR) is a transmembrane protein that is associated with cell survival, proliferation, and metastasis. In various infectious diseases, activated EGFR recruits leukocytes, leading to the production of antibacterial peptides, providing resistance against pathogens [15,16]. In addition, EGFR is highly developed in various solid tumors, and its role in several cancer cell lines has been identified [17]. Epidermal growth factor receptor was recently reported to play an important role in osteoblast differentiation in MC3T3-E1 cells [18]. According to previous reports, molecules downstream of EGFR, such as PI3K/Akt, play an important role in regulating HO-1 induction, [19] and the role of EGFR in CORM-2-induced HO-1 expression has been investigated. In addition, previous studies have demonstrated that Src family kinases (SFKs) act as targets of G protein-coupled receptors (GPCRs) in the trans-activation of EGFR through direct phosphorylation of the cytoplasmic domain of EGFR and play an important role in mediating HO-1 induction [20]. However, a role for HO-1 and EGFR in human periodontal ligament cells, in which periodontitis is induced, has not been identified.

*Panax ginseng* C.A. Meyer (Araliaceae) has been used widely as a traditional herbal medicine in Korea, Japan, and China. In addition, several ginsenosides present in *P. ginseng* exhibit anti-cancer, anti-inflammatory, antioxidant, and immunomodulatory activities [21,22,23]. Therefore, in this study, the main components obtained from *P. ginseng* fruit were identified, and the contents were evaluated using a validated analysis method. Few previous studies have investigated the ingredients of ginseng fruit or their activities. Here, the effects of each major component on inflammation in PG-LPS-stimulated human periodontal ligament cells and alveolar bone production were evaluated, which represent important treatment strategies for periodontitis.

## 2. Result

### 2.1. Structure and Specificity of Isolated Ginsenosides

**Figure 1:**
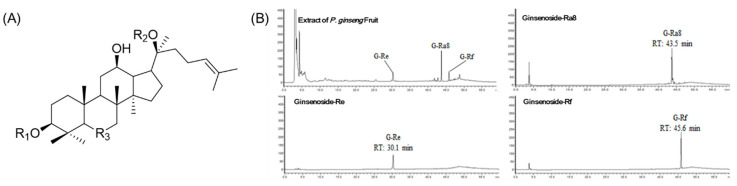
Structure of isolated ginsenosides and high-performance liquid chromatography (HPLC) chromatograms. The chemical structures of ginsenoside-Re, Ra8, and Rf (**A**). The analysis of *Panax ginseng* fruit extract and ginsenoside-Re, -Ra8, and -Rf using HPLC diode-array detector (DAD) (**B**). Diode array detector wavelength (203 nm).

To isolate ginsenoside, the extract of *Panax ginseng* fruits was sequentially partitioned with *n*-hexane, EtOAc, and *n*-BuOH, and six fractions were obtained from the *n*-BuOH fraction; the fractionation method is described in Section 4.2. The compounds **1**, **2**, and **3** obtained from the *n*-BuOH fraction were obtained from fractions 2, 3, and 4 and compared with the previously reported NMR spectra literature. They were identified as ginsenoside-Re (G-Re), ginsenoside-Ra8 (G-Ra8), and ginsenoside-Rf (G-Rf) (Figure 1A), (Table 1). The specificity of ginsenoside-Re, ginsenoside-Ra8 (G-Ra8), and ginsenoside-Rf was confirmed by comparing their retention times and wavelengths with those in the chromatograms of *P. ginseng* fruits extract. Ginsenoside-Re (RT: 30.1 min), G-Ra8 (RT: 43.5 min), and G-Rf (RT: 45.6 min) were confirmed at a UV wavelength of 203 nm; the compounds were not affected by repeated analysis; the purity of isolated compounds **1**, **2**, and **3** was 88%, 90%, and 92%, respectively (Figure 1B).

### 2.2. Linearity, Limit of Detection (LOD) and Limit of Quantitation (LOQ)

**Figure 2:**
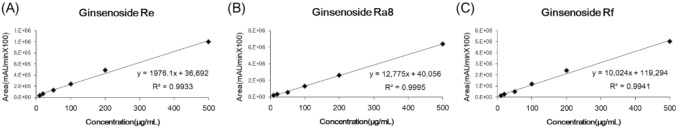
Calibration curves of G-Re (**A**), G-Ra8 (**B**), and G-Rf (**C**).

Based on the results of the analysis, a calibration curve for each standard substance was prepared with the peak area (mAU × 100) on the *y*-axis and the concentration of the standard solution (μg/mL) on the *x*-axis. A linear regression equation (*y* = a*x* + b) and correlation coefficient (*R*^2^) were determined. The *R*^2^ values of the three indicators were G-Re (0.9933), G-Ra8 (0.9995), and G-Rf (0.9941) (Figure 2), which showed good linearity (>0.99). Additionally, the limit of detection (LOD) and limit of quantitation (LOQ) were determined as follows: G-Re = 0.37 and 1.13 μg/mL; G-Ra8 = 0.69 and 2.08 μg/mL; and G-Rf = 0.23 and 0.70 μg/mL, respectively (Table 2).

### 2.3. Precision and Accuracy of the Analysis Method

Intra- and inter-day tests were performed based on the concentration at which linearity occurred, and the relative standard deviation (RSD %) was calculated to determine the precision. Variations are expressed as the RSD (%). In order to measure the precision and accuracy, G-Re, G-Ra8, and G-Rf were analyzed three times by preparing three quality control concentrations of 25, 125, and 250 μg/mL, respectively. Consequently, the RSD (%) of the intra-day test was within the range of 0.24–2.50% for G-Re, 0.56–0.27% for G-Ra8, and 0.12–1.75% for G-Rf. For the intra/inter-day test, the G-Re values ranged from 93.6% to 110.0%, G-Ra8 ranged from 101.0% to 102.9%, and G-Rf ranged from 89.7% to 108.8%. All three indicators were found to have excellent RSD (%) within 3.00% precision, and the accuracy of this method was good (Table 3).

### 2.4. Simultaneous Quantitative Analysis of G-Re, G-Ra8, and G-Rf Isolates from P. ginseng Fruit Extracts

The G-Re, G-Ra8, and G-Rf from *P. ginseng* fruit extract (PGFE) were quantitatively and simultaneously analyzed by applying previously validated methods. The results revealed that 1 g of PGFE contained about 1.01 ± 0.03 mg of G-Re, 0.33 ± 0.01 mg of G-Ra8, and 0.55 ± 0.04 mg of G-Rf (Table 4).

### 2.5. Effect of Ginsenoside G-Re, G-Ra8, and G-Rf on Human Periodontal Ligament (HPDL) Cell Viability and Confluence

**Figure 3:**
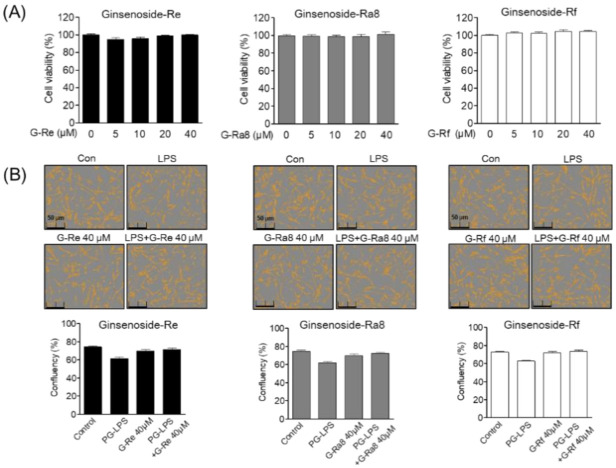
Effects of ginsenosides on human periodontal ligament (HPDL) cells’ viability and confluency. The HPDL cells were incubated 1 × 10^4^ cell/mL for 24 h, then after treatment with each indicated concentration of ginsenosides for 24 h, cell viability was analyzed through an MTT assay (**A**). Cell confluency was determined using the Incucyte^®^ Live-Cell assay system to mark the cell confluency of normal cells (**B**).

To determine the cytotoxic potential of PGFE, G-Re, G-Rc, G-Ra8, and G-Rf, their effects on HPDL cell viability were evaluated. Cell viability was calculated by comparing the ratio of the control cell group to the treated cell groups; the viability of the control cell group was taken as 100%. Human periodontal ligament cells were treated with different concentrations of each ginsenosides (5–40 μM) for 24 h. The results showed that ginsenosides was not toxic at concentrations between 5 and 40 μM (Figure 3A). In addition, ginsenosides and PG-LPS had no effect on the confluency of the HPDL cells (Figure 3B).

### 2.6. Ginsenosides Induces HO-1 Expression by Promotion of Nrf2 in HPDL Cells

**Figure 4:**
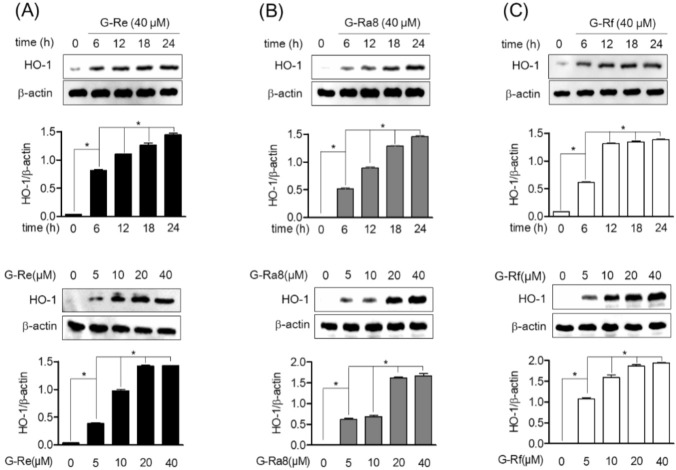
Effect of ginsenosides on heme oxygenase (HO)-1 induction in HPDL cells. The cells (1 × 10^6^ cells/mL) were treated with 40 μM for the indicated time (0, 6, 12, 18, and 24 h) or with the indicated concentrations of each ginsenosides Re (**A**), Ra8 (**B**), and Rf (**C**) (5, 10, 20, and 40 μM) for 6 h. The expression of HO-1 was determined by Western blot analysis. * *p* < 0.05 was considered as significant differences between each treated group.

**Figure 5:**
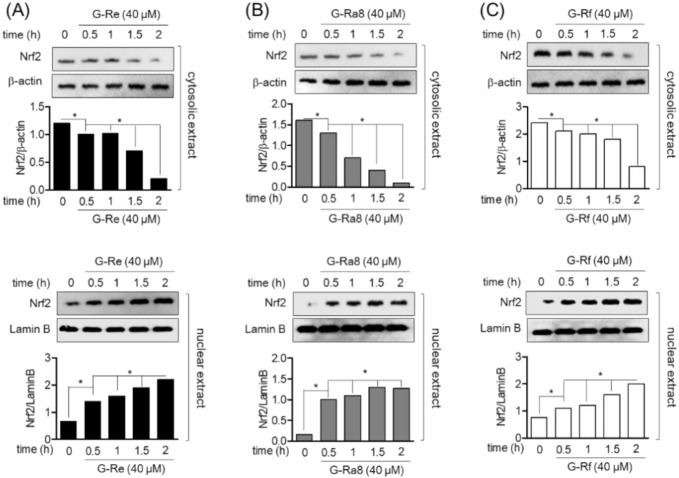
The nuclear translocation of nuclear factor-red blood cell 2-related factor 2 (Nrf2) was treated with ginsenoside Re (**A**), Ra8 (**B**), and Rf (**C**) according to the indicated concentration (40 μM) and time (0.5–2 h) and analyzed by Western blot. * *p* < 0.05 was considered as significant differences between each treated group.

Heme oxygenase 1 is induced as a defense mechanism against various inflammatory responses and is induced by the translocation of Nrf2. Therefore, in this study, the induction of HO-1 expression by ginsenosides, and Nrf2 translocation to the nucleus in human periodontal ligament cells were evaluated. The HPDL cells were treated with ginsenoside Re, Ra8, and Rf 40 μM of for 6, 12, 18, and 24 h, and the protein expression of HO-1 was evaluated by Western blot analysis. The expression of HO-1 increased with time, and the highest expression was observed at 24 h. In addition, ginsenoside Re (Figure 4A), Ra8 (Figure 4B), and Rf (Figure 4C) increased the expression of HO-1 in a concentration-dependent manner over 5–40 μM. The role of ginsenosides on Nrf2 translocation for the regulation of HO-1 protein and gene expression was evaluated. Treatment of HPDL cells with ginsenosides Re (Figure 5A), Ra8 (Figure 5B), and Rf (Figure 5C) at 40 μM reduced the cytosolic concentration of Nrf2 in a time-dependent manner from 0.5 to 2 h and increased the amount of Nrf2 nuclear translocation. Therefore, the induction of HO-1 expression through the nuclear translocation of Nrf2 by ginsenosides is expected to exert anti-inflammatory effects through cellular protective mechanisms.

### 2.7. The Regulatory Effect of Ginsenosides on EGFR Expression

**Figure 6:**
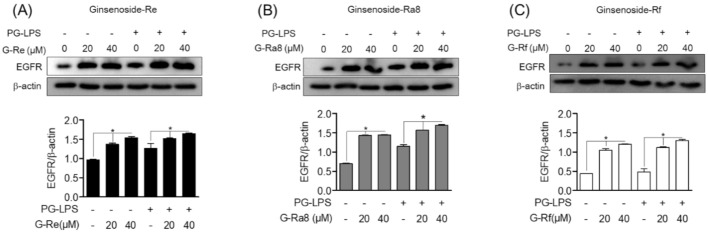
The regulation effect of EGFR protein by ginsenosides. The HPDL cells (1 × 10^6^ cells/mL) were pre-treated with each ginsenosides: Re (**A**), Ra8 (**B**), and Rf (**C**) (20 μM, 40 μM) at the indicated concentration for 12 h, followed by treatment with PG-LPS for 24 h, and the expression of epidermal growth factor receptor (EGFR) protein was measured through Western blot analysis. * *p* < 0.05 was considered as significant differences between each control group.

To investigate the role of EGFR signaling in the regulation of HO-1 expression, we examined whether EGFR is regulated by ginsenosides. First, HPDL cells were treated with ginsenosides at 20 and 40 μM for 12 h. Then, PG-LPS-stimulated cells were divided into groups, which were treated or untreated. Cells were then treated for 24 h and EGFR protein expression was subsequently evaluated by Western blot analysis. Ginsenoside-Re (Figure 6A), -Ra8 (Figure 6B), and -Rf (Figure 6C) increased EGFR protein expression compared to the control group, and similar results were confirmed in the PG-LPS group. These results suggest that ginsenosides plays an important role in the regulation of EGFR protein expression, in addition to the regulation of HO-1 expression via nuclear translocation of Nrf2 in HPDL cells.

### 2.8. Induction of HO-1 Protein by Ginsenosides Is Regulated by EGFR

**Figure 7:**
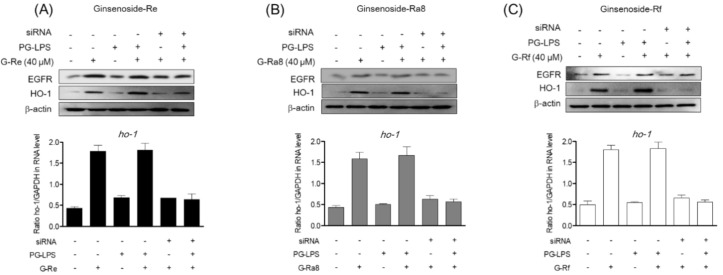
The HO-1 protein expression by ginsenoside is regulated through EGFR. The HPDL cells (5 × 10^4^ cells/mL) were cultured for 24 h and then treated with 100 pmol of EGFR siRNA for 24 h to induce EGFR silencing. Thereafter, each ginsenoside-Re (**A**), -Ra8 (**B**), and -Rf (**C**) were treated at the indicated concentration for 6 h, and then PG-LPS was treated for 24 h. After, the protein amount and gene level were measured using Western blot analysis and real time PCR analysis.

In a previous study, ginsenoside-Re, -Ra8, and -Rf were shown to induce the expression of HO-1 via the nuclear translocation of Nrf2, and a regulatory role for EGFR was reported. Recent studies have investigated the role of EGFR in the regulation of HO-1 expression. However, the role of EGFR in the regulation of HO-1 expression in HPDL cells and periodontitis has not been confirmed. Therefore, we evaluated the role of EGFR in the expression of HO-1 by ginsenosides in HPDL cells. In this study, we investigated the effect of EGFR silencing on HO-1 expression. Expression of HO-1 and EGFR proteins was induced by treatment with ginsenoside-Re (Figure 7A), -Ra8 (Figure 7B), and -Rf (Figure 7C), and similar levels of protein were found in the group treated with PG-LPS. However, when EGFR was silenced by siRNA, HO-1 expression was also reduced. In addition, real-time PCR was used to evaluate the effect of EGFR silencing by siRNA on HO-1 gene expression. The results showed that the gene expression of HO-1 was similar to those obtained for the protein expression of HO-1. These results suggest that the induction of HO-1 expression by ginsenosides is regulated by EGFR.

### 2.9. Ginsenoside Inhibits PG-LPS-Induced Pro-Inflammatory Mediator Production in HPDL Cells

**Figure 8:**
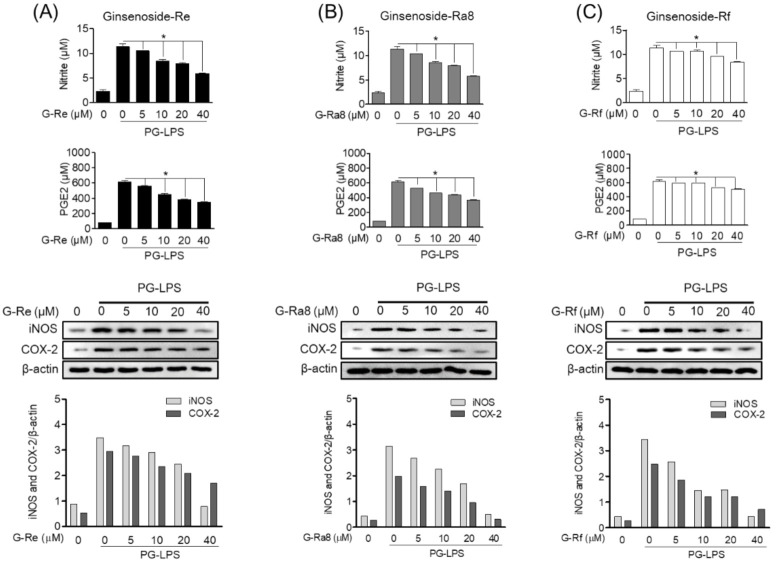
Ginsenosides inhibit PG-LPS-induced pro-inflammatory mediator production in HPDL cells. HPDL cells (1 × 10^6^ cells/mL) were cultured for 24 h and then treated with ginsenoside-Re (**A**), -Ra8 (**B**), and -Rf (**C**) at the indicated concentrations for 6 h. Thereafter, PG-LPS (1 μg/mL) was treated for 24 h, and the expression levels of inflammatory mediator Western blot and ELISA kit. * *p* < 0.05 was considered as significant differences between PG-LPS-treated groups.

To evaluate the inhibitory effect on periodontitis, the effects of ginsenoside-Re, -Ra8, and -Rf on the expression of pro-inflammatory cytokines in HPDL cells stimulated with PG-LPS were investigated. Prostaglandin E2 (PGE2), induced by cyclooxygenase (COX)-2, and nitric oxide (NO), induced by inducible nitric oxide synthase (iNOS), are important for assessing the level of inflammation. In the present study, ginsenoside-Re (Figure 8A), -Ra8 (Figure 8B), and -Rf (Figure 8C) inhibited the protein expression of the pro-inflammatory mediators iNOS and COX-2 in PG-LPS-stimulated HPDL cells in a concentration-dependent manner. This inhibitory effect was most effective in the group treated with 40 μM ginsenosides. In addition, suppressed production of these pro-inflammatory mediators effectively regulated the production of PGE2 and NO. Ginsenosides-Re, -Ra8, and -Rf exerted anti-inflammatory effects in an in vitro model of HPDL periodontitis stimulated by PG-LPS, suggesting a potential role for ginsenosides in alleviating periodontitis.

### 2.10. HO-1 Induced by Ginsenosides in PG-LPS-Stimulated HPDL Cells Regulates Pro-Inflammatory Cytokines

**Figure 9:**
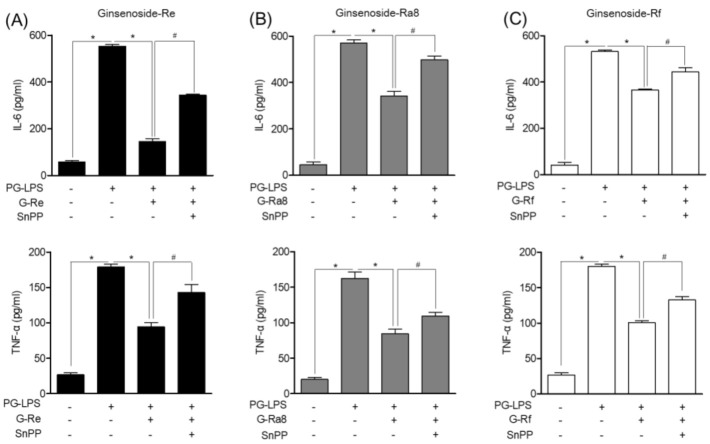
The HO-1 induced by ginsenosides in HPDL cells stimulated with PG-LPS regulates pro-inflammatory cytokines. HPDL cells (1 × 10^6^ cells/mL) were cultured for 24 h and then treated with or without protoporphyrin IX (SnPP) and ginsenoside-Re (**A**), -Ra8 (**B**), and -Rf (**C**) at the indicated concentrations (5–40 μM) for 6 h. Thereafter, PG-LPS (1 μg/mL) was treated for 24 h, and the expression levels of pro-inflammatory cytokines were analyzed using an ELISA kit. * *p* < 0.05 was considered significant for the PG-LPS treated groups. # *p* < 0.05 was considered significant between each ginsenoside+ PG-LPS-treated group.

Previous studies have shown that ginsenosides inhibit the production of pro-inflammatory mediators. Therefore, the effects of ginsenosides on IL-6 and TNF-α, the major inflammatory cytokines involved in periodontitis, and the role of HO-1 were evaluated. Tin protoporphyrin IX (SnPP) is an HO-1 inhibitor. Here, we inhibited HO-1 expression with SnPP and evaluated the effect on the inflammatory cytokines involved in periodontitis. The PG-LPS-induced inflammatory cytokine production decreased following treatment with ginsenoside-Re (Figure 9A), -Ra8 (Figure 9B), and -Rf (Figure 9C). Treatment with the HO-1 inhibitor SnPP confirmed that the level of inflammatory cytokine production was restored. Therefore, it is possible that the induction of HO-1 by ginsenosides inhibits pro-inflammatory cytokine expression. The anti-inflammatory effect of ginsenosides and their potential in the treatment of periodontitis have been suggested based on the results of an in vitro model stimulated with PG-LPS.

### 2.11. Effect of Ginsenosides Though EGFR-Mediated HO-1 Expression on Pro-Inflammatory Cytokines

**Figure 10:**
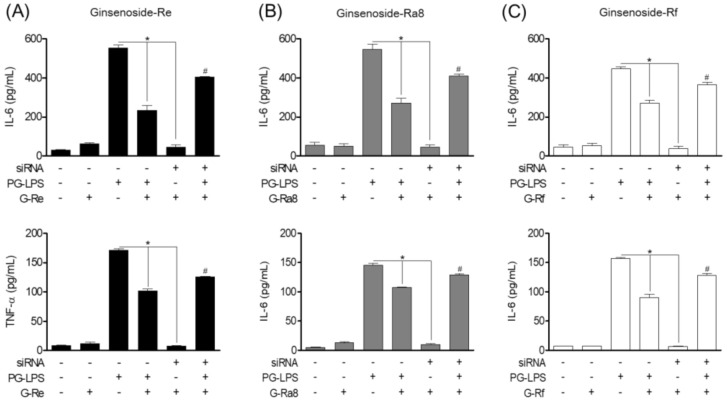
Effect of ginsenosides induced regulation of HO-1 expression in EGFR on pro-inflammatory cytokines. Ginsenosides-Re (**A**), -Ra8 (**B**), and -Rf (**C**) were treated with the concentrations indicated for each group with or without EGFR silenced, followed by PG-LPS (1 μg/mL) treatment for 24 h. After that, the production of pro-inflammatory cytokines was measured using an ELISA kit. * *p* < 0.05 was considered significant differences between siRNA-treated groups. * *p* < 0.05 was considered significant between PG-LPS-treated groups. # *p* < 0.05 was considered significant between each ginsenoside+ siRNA-treated group.

Previous studies have shown that ginsenosides inhibit the production of pro-inflammatory mediators and cytokines through HO-1 expression. Therefore, to investigate the role of EGFR in controlling pro-inflammatory cytokine production through HO-1 of ginsenosides, we silenced EGFR signaling using siRNA and evaluated the effect of ginsenosides on the pro-inflammatory cytokines IL-6 and TNF-α. The results showed that PG-LPS treatment increased the production of the pro-inflammatory cytokines IL-6 and TNF-α, and that this was effectively reduced following treatment with ginsenosides-Re, -Ra8, and -Rf. However, silencing the EGFR signal with EGFR siRNA confirmed the inhibitory effect of the pro-inflammatory cytokines IL-6 and TNF-α by ginsenoside-Re (Figure 10A), -Ra8 (Figure 10B), and- Rf (Figure 10C) was reversed. 

### 2.12. Effects of HO-1 Expression Induced by Ginsenosides on Osteoblast Differentiation and Osteoblast-Specific Gene Expression

**Figure 11:**
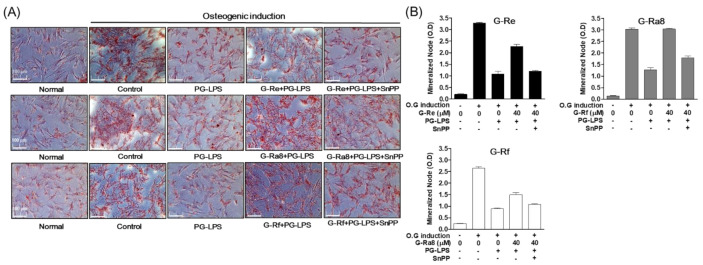
Effect of osteogenic induction through by HO-1 expression. The HPDL cells (5 × 10^3^ cells/mL) were pretreated with or without the indicated concentration of tin protoporphyrin IX (SnPP), then after, each ginsenoside-Re, Ra8, and -Rf was treated for 6 h at the indicated concentration, and then incubated with PG-LPS for 14 days. The result of mineralization was measured by Alizarin Red S (ARS) (**A**) staining and mineralization node (**B**).

**Figure 12:**
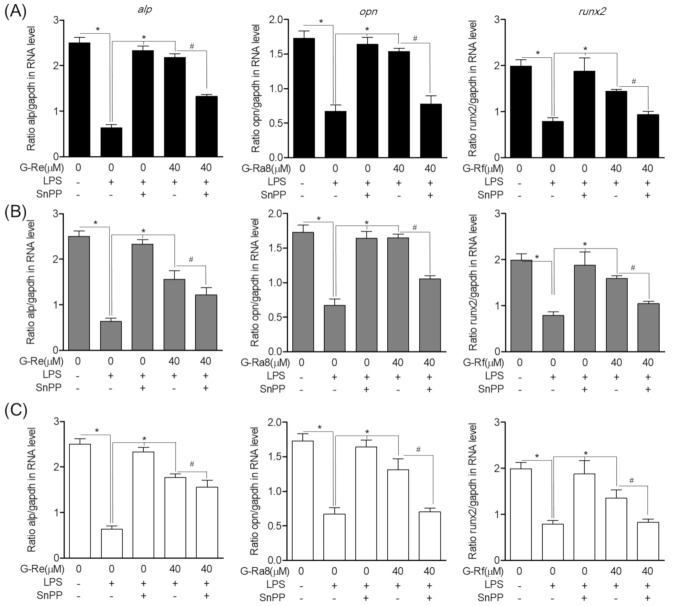
The HO-1 expression by ginsenosides regulates the level of osteoblast-specific genes. The HPDL cells (5 × 10^3^ cells/mL) were pretreated with or without the indicated concentration of tin protoporphyrin IX (SnPP), then, each ginsenoside-Re (**A**), -Ra8 (**B**), and -Rf (**C**) was treated for 6 h at the indicated concentration and then incubated with PG-LPS for 7 days. The level of osteogenic specific genes *alp*, *opn*, and *runx2* were confirmed by real-time PCR. The results were normalized to *gapdh* expression. * *p* < 0.05 was considered significant between PG-LPS-treated group. # *p* < 0.05 was considered significant between each ginsenoside+ PG-LPS-treated groups.

In addition to the anti-inflammatory effect of ginsenosides via the regulation of HO-1 expression in PG-LPS-stimulated HPDL cells, we also evaluated the effect of ginsenoside-induced HO-1 expression on other important treatment strategies for periodontitis such as the inhibition of alveolar bone loss. To induce osteoblast differentiation, HPDL cells were incubated in osteoblast differentiation media for 7 days. We found that the intensity of Alizarin Red S (ARS) staining was weaker in the group treated with PG-LPS compared with that in the control group in terms of the induction of differentiation. In addition, the level of staining was recovered following treatment with ginsenoside-Re, -Ra8, and -Rf. The effect of ginsenoside-Re, -Ra8, and -Rf on osteoblast differentiation was inhibited following treatment with SnPP, an inhibitor of HO-1 (Figure 11A). The effects of Re, Ra8, and Rf on osteoblast differentiation were similarly confirmed based on the level of mineralization (Figure 11B). In addition, we evaluated the effect of ginsenoside-induced HO-1 expression on the expression of osteoblast-specific genes alp, opn, and runx2 that play important roles in early osteoblast formation. Ginsenoside-Re (Figure 12A), -Ra8 (Figure 12B), and -Rf (Figure 12C) induced the recovery of osteoblast-specific genes repressed by PG-LPS. As observed for osteoblast formation, the expression of osteoblast-specific genes was inhibited by the HO-1 inhibitor SnPP. These results suggest that HO-1, induced by ginsenosides, inhibits periodontal inflammation and the loss of osteoblasts, thereby exerting a protective effect on osteoblasts.

## 3. Discussion

*Panax ginseng* has long been used as a medicinal herb in traditional Chinese and Korean medicine [22], and ginsenoside are its main pharmacological components. These belong to triterpenoid saponins and are composed of a dammarane skeleton (17 carbons in a four-ring structure) with various sugar moieties (e.g., glucose, rhamnose, xylose, and arabinose) attached to the C-3 and C-20 positions [24]. According to a previously reported study on the composition and content of ginseng using HPLC-DAD, various ginsenosides contained in ginseng were detected at a wavelength of 203 nm, and the contents of various ginsenosides were quantified from the extract of ginseng [24]. However, while studies have investigated many of these ginseng components, the main components of ginseng fruit and the ginsenoside contents have not been accurately determined. In this study, the contents of ginsenoside-Re, -Ra8, and -Rf isolated from ginseng fruit extract were measured using a simultaneous analysis method. The *P. ginseng* extract was found to contain approximately 1.01 ± 0.03 mg of G-Re, 0.33 ± 0.01 mg of G-Ra8, and 0.55 ± 0.04 mg of G-Rf in 1 g. In addition, the effects of three ginsenosides isolated from standardized ginseng fruit extracts were evaluated in an in vitro periodontitis model.

Epidermal growth factor receptor signaling promotes bone formation by inducing bone homeostasis, osteoblast proliferation, and differentiation of osteoblasts such as MC3T3-E1. Recent studies have reported it to exert cytoprotective effects through anti-inflammatory and anti-apoptotic properties. In addition, since the activity of EGFR affects the induction of Nrf-2/HO-1, it is considered to be important in the regulation of osteoblast inflammation by downregulating the levels of IL-1 and IL-6 [18]. However, the role of EGFR-mediated HO-1 in the induction of periodontitis in HPDL cells stimulated with PG-LPS has not yet been investigated. The results of our study showed that ginsenoside-Re, -Ra8, and -Rf induced nuclear the translocation of Nrf2, inducing HO-1 expression, and inhibiting the production of pro-inflammatory mediators. In particular, ginsenosides inhibited the release of the pro-inflammatory cytokines, TNF-α and IL-6, which are important inflammatory factors in periodontitis [25]. We investigated the relationship between these anti-inflammatory effects and HO-1 following SnPP treatment and confirmed that the anti-inflammatory effect was reversed when HO-1 expression was inhibited. In addition, the results confirmed that ginsenosides exerted an anti-inflammatory effect through HO-1, and by inhibiting alveolar bone loss, which is an important treatment strategy for periodontitis. The anti-inflammatory effect of HO-1 in osteoblasts regulates the expression of specific genes by protecting osteoblast proliferation [26]. In this study, the ginsenoside-induced HO-1-induced osteoblast proliferation. Notably, the levels of osteoblast-specific genes alp, opn, and runx2, which were lost following treatment with PG-LPS, were restored. A recent study reported that the induction of EGFR-mediated carbon monoxide-releasing molecule-2 (CORM-2) in human bronchial smooth muscle cells promotes HO-1 expression, whereas in glioblastoma, the phosphoinositide 3-kinase (PI3K) signaling pathway is activated through the binding of EGFR and EGF to induce HO-1 expression [19,20,27]. Therefore, to confirm the role of EGFR in the inhibition of periodontitis and alveolar bone loss following induction of HO-1 expression by ginsenosides, EGFR was silencing with EGFR siRNA. We found that the expression of HO-1 was regulated by EGFR silencing, and the inhibitory effects of periodontitis and alveolar bone loss were reversed.

To our knowledge, no previous studies have reported the components and contents of major ginsenosides in *P. ginseng* fruit, while several studies have reported the correlation between EGFR and HO-1 or various pharmacological protective activities associated with the regulation of HO-1 in each EGFR, but its role in periodontitis has not yet been clarified. Therefore, in this study, the pharmacological effect on periodontitis was demonstrated by analyzing the contents of ginsenoside-Re, -Ra8, and -Rf isolated from *P. ginseng* fruit.

## 4. Materials and Methods

### 4.1. Chemicals and Reagents

3-(4,5-Dimethylthiazol-2-yl)-2,5-diphenyltetrazoliumbromide (MTT) was purchased from Sigma–Aldrich (Saint Louis, MO, USA). Dulbecco’s modified Eagle’s medium (DMEM), minimum essential medium alpha (α-MEM), and fetal bovine serum (FBS) were purchased from Welgene Bioscience (Daegu, Korea). Primary antibodies for iNOS, COX-2, HO-1, and EGFR rabbit polyclonal antibodies were obtained from Cell Signaling Technology Inc. (Danvers, MA, USA). The enhanced chemiluminescence (ECL) Western blotting detection system was obtained from Advansta Inc. (San Jose, CA, USA). The HPLC-grade acetonitrile and water were obtained from Honeywell-Burdick and Jackson (Muskegon, MI, USA). The TNF-α and IL-6 ELISA (enzyme-linked immunosorbent assay) kits were purchased from R&D Systems (Minneapolis, MN, USA). Lipopolysaccharide isolated from *P. gingivalis* (PG-LPS) was purchased from InvivoGen (San Diego, CA, USA). Alizarin Red S and tin protoporphyrin IX (SnPP) were obtained Sigma–Aldrich (St. Louis, MO, USA). *n*-Hexane, ethyl acetate, *n*-butanol, chloroform, and MeOH were purchased from Daejung Chemicals and Metals CO. LTD. (Siheung, Korea).

### 4.2. Extraction of Plant Material and Ginsenosides Isolation

The fruits of *P. ginseng* were cultivated and harvested in Gyeonggi Province Anseong (Anseong, Korea) and provided by the national agricultural cooperative federation of Gyeonggi province Anseong. The fruits of *P. ginseng* extract were provided by Korea Ginseng Corporation (KGC) LIFENGIN INC. (Seoul, Korea) The preparation of *P. ginseng* fruit extract was enzymatically decomposed (amylase, pectinase, cellulose) at 50 °C for 4 h, which was followed by enzyme inactivation at 90 °C for 10 min. Then, it was prepared by filtering perlite and then vacuum concentration. Then, the concentrated extract (240.1 g) was suspended in water and successively partitioned using *n*-hexane, ethyl acetate, and *n*-butanol. The butanol extract (45.5 g) was isolated using silica gel column chromatography with CHCl_3_:MeOH (10:2, 10:2.5, 10:3, 10:4, and 10:5) as solvent, and six fractions were obtained (fractions 1–6). Among them, fraction 2 (1.7 g), 3 (1.1 g), and 4 (1.8 g) were obtained, and each fraction was purification by performing a column chromatogram (Sephadex LH-20, MeOH). Thereafter, G-Re (14.0 mg) at fraction 2, G-Ra8 (12.8 mg) at fraction 3, and G-Rf (12.1 mg) at fraction 4 were obtained. The purity of the isolated ginsenosides was evaluated based on 100% of the total peak area values obtained from each HPLC-DAD (wavelength 203 nm) chromatogram, and the area values of the remaining ginsenosides, excluding the peak area values of impurities, were calculated as percentages. It was identified as 88% for ginsenoside-Re, 90% for ginsenoside-Ra8, and 92% for ginsenoside-R. The isolated ginsenosides were measured for mass analysis by liquid chromatography-mass spectrometry (LC-MS, Agilent 6120 single quadrupole model, Santa Clara, CA, USA), and the structure was identified by nuclear magnetic resonance (NMR, JNM-ECZR 500 MHz, Tokyo, Japan). The isolated ginsenosides was identified by comparison with previously reported literature [24,28].

### 4.3. High-Performance Liquid Chromatography (HPLC) Analysis

The fractions were purified using semi-preparative HPLC using a Phenomenex C18 column (4.6 × 250 mm, 5 μm I.D., Torrance, CA, USA). The column temperature was 30 °C. These separations were achieved using the following gradient program with water (A) and acetonitrile (B): 0 min (17% B), 20 min (25% B), 38 min (42% B), and 60 min (17% B). The flow rate was set at 0.8 mL/min and the sample volume used for injection was 10 μL. Ginsenosides were detected using a diode array detector at 203 nm. The HPLC conditions for quantitative analysis are specified in Table 5.

### 4.4. Validation of HPLC Analysis Method

The 1 mg/mL standard solution was prepared by dissolving 1 mg of each indicator (G-Re, G-Ra8, and G-Rf) in water. Then, the solution was serially diluted and used to prepare a calibration curve. For linearity evaluation, G-Re, G-Ra8, and G-Rf were dissolved in water and mixed and serially diluted to yield concentrations of 10, 20, 50, 100, 200, and 500 μg/mL for HPLC analysis. Based on the results, calibration curves for the three standards were prepared. The calibration curves were written in the following format: y = ax + b (a: slope of calibration curve, b: y-intercept, x: sample concentration, y: area of peak); correlation coefficient (*R*^2^) was obtained using the equation. The linearity was evaluated using the *R*^2^ value of the calibration curve.

### 4.5. Inter-Day and Intra-Day Tests for Method Validation

To check the minimum detectable concentration and the minimum quantifiable concentration of the analysis, the limit of detection (LOD) and limit of quantitation (LOQ) were measured using the following equations: LOD = 3.3 × (σ/S), LOQ = 10 × (σ/S) (σ: standard deviation, S: slope of calibration curve). To demonstrate the feasibility of quantitative analysis, three indicators were subjected to repeated experiments. Precision was evaluated using the relative standard deviation (RSD%); RSD% value was judged to have excellent precision within 3%. The intra-day test result was evaluated using the RSD% of the three concentrations, where linearity was confirmed during three repeated measurements. The inter-day test was evaluated by calculating the RSD% using results of the repeated measurements (three times) at the above three concentrations.

### 4.6. Preparation of Human Periodontal Ligament Cells

The HPDL cells were obtained from the third molar of each donor as previously described [29]. The protocols for the isolation and culture of HPDL (human periodontal ligament) cells were reviewed and approved by the Institutional Review Board of Kyungpook National University (Daegu, Korea) (KNU 2017-78). A brief description of the culture of HPDL cells is as follows. The HPDL cells were cultured in α-MEM supplemented with 10% (*v*/*v*) fetal bovine serum (FBS), and 1% penicillin/streptomycin (Gibco BRL, Grand Island, NY, USA) and cultured at 37 °C in a humidified atmosphere with 5% CO_2_.

### 4.7. The Cell Viability and Coefficient Assays of HPDL Cells

The effects of ginsenosides isolated (G-Re, G-Ra8, and G-Rf) on HPDL cells viability were determined using an MTT assay. The HPDL cells (1 × 10^4^ cells/well) were seeded into a 96-well plate at 37 °C in a humidified atmosphere (5% CO_2_) for the 24 h. Next, they were treated with G-Re, G-Ra8, and G-Rf (5, 10, 20, and 40 μM) before incubating at 37 °C in humidified atmosphere (5% CO_2_) for 24 h. MTT (5 mg/mL) solution was added before incubating for another 3 h. After the incubation, the supernatant was removed and 150 μL of dimethyl sulfoxide (DMSO) was added. The optical density was measured at 595 nm using a microplate reader (Tecan Trading AG) (Männedorf, Switzerland). In addition, the count of cells was measured using Incucyte^®^ Live-Cell analysis systems (Göttingen, Germany).

### 4.8. Nitric Oxide and Cytokines Production Assay

The HPDL cells (1 × 10^4^ cells/well) were seeded into a 96-well plate at 37 °C in humidified atmosphere (5% CO_2_) for the 24 h. Next, they were treated with various concentrations of ginsenoside Re, Ra8, and Rf (5, 10, 20, and 40 μM) before incubating for 2 h. After that, they were treated with PG-LPS (1 μg/mL) and incubated for 24 h. After the incubation, 100 μL of supernatant was mixed with 100 μL Griess reagent (1% sulfanilamide, 2.5% phosphoric acid containing 0.1% naphthylethylenedi-amine) and allowed to react for 10 min. Then, nitrite production was determined by measuring the absorbance at 540 nm using a NaNO_2_ standard curve. In addition, the production of TNF-α, IL-6 and PGE_2_ was analyzed using the kit (R&D Systems, Minneapolis, MN, USA), and each ginsenosides was treated and incubated for 6 h, and then PG-LPS was treated for 18 h and then analyzed from the obtained supernatant.

### 4.9. Evaluation of Mineralization Analysis by Ginsenosides

The HPDL cells were cultured in a 24-well culture plate at 5 × 10^3^ cells/well, and then in osteo-induction medium supplemented with 50 μg/mL ascorbic acid, 0.1 μM dexamethasone, and 10 mM β-glycerophosphate for 14 days to induce bone formation. After that, each group was pretreated as described in the figure legend, and when mineralized nodules were formed, the mineralized cells were fixed with 4% polyformaldehyde for 30 min, stained with 0.1% Alizarin Red S (Sigma–Aldrich, St. Louis, MO, USA), and at room temperature at pH 4.3 for fixed 30 min. Then, to measure the content of calcium precipitate, the cetyl pyridine chloride (CPC) method was applied, and the absorbance at 560 nm was measured using a multifunctional microplate reader (M1000 Pro, TECAN, Männedorf, Switzerland).

### 4.10. Western Blot Analysis

The HPDL cells (1 × 10^5^ cells/well) were seeded into 6-well plates and incubated overnight at 37 °C in humidified atmosphere (5% CO_2_). Then, they were treated as described above. The treated cells were harvested, lysed in RIPA buffer (50 mM Tris pH 8.0, 150 nM NaCl, 0.02% sodium azide, 0.2% SDS, 1 M MPMFS, 10 μL/mL aprotinin, 1% igapel 630 (Sigma-Aldrich, USA), 10 nM NaF, and 0.5 nM EDTA) and centrifuged at 23,000× *g* for 15 min. Equal amounts of proteins were subjected to 10% SDS-polyacrylamide gel electrophoresis and electro-transferred to polyvinylidene fluoride membranes (0.45 μm). After blocking with 5% non-fat milk for 1 h, the membranes were probed using β-actin, iNOS, COX-2, HO-1, and EGFR (Cell Signaling Technology Inc., Danvers, MA, USA) at 4 °C overnight. The next day, the membranes were washed with Tris-buffered saline with 0.1% Tween^®^ 20 Detergent buffer and blotted with anti-rabbit or anti-mouse secondary antibody (Cell Signaling Technology Inc.) for 2 h. After washing, the proteins were activated using an ECL (Enhanced Chemiluminescence) detection kit and determined using enhanced chemiluminescence with an ImageQuant LAS 4000 bio-molecular imager (GE Healthcare Life Sciences, Marlborough, MA, USA).

### 4.11. Real-Time Quantitative PCR

The expression levels of alp, opn, RUNX2, and HO-1 were detected by RT-PCR. The RNA was extracted from the cell lysate using TRIZOL reagent, and the concentration of mRNA was analyzed by NanoDrop (Thermo Scientific, Waltham, MA, USA). After that, cDNA was synthesized using TOPscript™ RT DryMIX (dT18 plus). Real-time PCR reactions were run on a LightCycler 480 (Roche, Basel, Switzerland) instrument using TB Green^®^ Premix Ex Taq™ II (Tli RNaseH Plus). The mRNA level of each gene was normalized to GAPDH using glyceraldehyde-3-phosphate dehydrogenase (GAPDH), and all gene expression changes were analyzed three times. The analysis result was calculated using the following equation. 2−ΔΔCT, where ΔΔCT = (CT*target* − CT*gapdh*) at time *x* − (CT*target* − CT*gapdh*) at time 0, time x represents any time point, and time 0 represents the 1 × expression of the gene in the untreated cells normalized to *gapdh*. The primers are presented in Table 6.

### 4.12. Small Interfering RNA (siRNA)-Induced EGFR Gene Silencing

The SiRNA against EGFR was performed according to the transfection protocol of Lipofectamine 2000 (Invitrogen, Carlsbad, CA, USA). The HPDL cells were plated on 6-well plates with 5 × 10^3^ cells/mL in 1 mL culture medium. The EGF SiRNA (Santa-cruz, TX, USA) transfections were performed according to the manufacturer’s instructions of Lipofectamine 2000. The cells were then harvested for further experiments after re-incubating for 6 h in 37 °C of humidified atmosphere 5% CO_2_. The extent of gene knockdown was determined by Western blot.

### 4.13. Statistical Analysis

All experiments were conducted in triplicates. The data are presented as means ± standard deviation (SD). Independent samples were compared using Tukey’s test and the Student’s *t*-test using Sigma Plot software 12.1. *p*-Values < 0.05 were considered statistically significant.

## 5. Summary and Conclusions

In this study, the major ginsenosides of *P. ginseng* fruit, the components and contents of which have not yet been reported, were quantitatively analyzed through a validated simultaneous analysis method utilizing HPLC-DAD. In addition, in an in vitro model of PG-LPS-induced periodontitis, major ginsenosides demonstrated the effect of controlling EGFR-mediated HO-1 on the inhibitory effect of periodontal inflammation and alveolar bone loss, an important treatment strategy for periodontitis. Therefore, these data are of value for future research on the ingredients of *P. ginseng* fruit and indicate that the isolated ginsenoside-Re, -Ra8, and -Rf may have potential as future treatment options for periodontitis.

## Figures and Tables

**Table 1:** Structure of isolated ginsenosides (G).

Compounds	R1	R2	R3
**1** (G-Re)	H	O-Glc	Glc(2,1)Rha
**2** (G-Ra8)	Glc(2,1)Glc-4-butenoly	-	Glc(6,1)Ara(f)
**3** (G-Rf)	H	H	Glc(2,1)Glc

**Table 2:** Regression equation, limit of detection (LOD), and limit of quantitation (LOQ) of G-Re, G-Ra8, and G-Rf.

Linear Range (μg/mL)	Compounds	Regression Equation	*R* ^2^	LOD (μg/mL)	LOQ (μg/mL)
10–500	**1** (G-Re)	y = 1976.1x + 36,692	0.9933	0.37	1.13
	**2** (G-Ra8)	y = 12,775x + 40,056	0.9995	0.69	2.08
	**3** (G-Rf)	y = 10,024x + 119,294	0.9941	0.23	0.70

**Table 3:** Intra-day and Inter-day test (precision and accuracy measurement).

Compounds	Concentration (μg/mL)	Intra-Day (*n* = 3)			Inter-Day (*n* = 3)		
		Mean ± SD	RSD (%)	Accuracy	Mean ± SD	RSD (%)	Accuracy
G-Re	25	23.41 ± 0.58	2.50	93.6	24.03 ± 0.22	0.92	96.1
	125	128.56 ± 2.92	2.27	102.8	126.88 ± 1.46	1.15	101.5
	250	252.11 ± 1.42	1.16	100.8	249.08 ± 0.29	0.12	99.6
	25	24.69 ± 0.58	2.37	98.8	25.06 ± 0.22	0.88	100.2
G-Ra8	125	136.61 ± 1.21	0.96	101.3	126.24 ± 1.46	1.16	101.0
	250	228.33 ± 1.20	0.52	91.3	224.16 ± 0.29	0.13	89.7
	25	27.43 ± 0.27	1.23	109.7	27.49 ± 0.07	0.24	110.0
G-Rf	125	127.33 ± 2.01	0.59	101.9	128.65 ± 0.76	0.56	102.9
	250	272.09 ± 4.75	1.75	108.8	265.50 ± 3.49	1.31	106.2

Photo diode array detector method for the determination of G-Re, G-Ra8, and G-Rf (*n* = 3). RSD, relative standard deviation.

**Table 4:** The contents of G-Re, G-Ra8, and G-Rf in *P. ginseng* fruit extracts (*n* = 3).

Sample	Ginsenoside-Re	Ginsenoside-Ra8	Ginsenoside-Rf
Contents (mg/g)	1.01 ± 0.03	0.33 ± 0.01	0.55 ± 0.04

**Table 5:** The HPLC conditions for quantitative analysis.

Parameters	Conditions		
Analytical column	Phenomenex C18 (4.6 × 250 mm)		
HPLC detector	Diode array detector (203 nm)		
	Solvent A: Water Solvent B: ACN		
Mobile phase	Final time	Solvent	
	(min)	A (%)	B (%)
	0	83	17
	20	75	25
	38	58	42
	60	83	17
Flow rate		0.8 mL/min	
Column oven temperature		30 °C	
Injection volume		10 μL	
Run time		60 min	

**Table 6:** Primer sequences.

Target Gene	Sequence (5′→3′)		Accession Number
*ho-1*	Forward	CCAGGCAGAGAATGCTGAGTTC	NM_002133
	Reverse	AAGACTGGGCTCTCCTTGTTGC	
*alp*	Forward	TGCAGTACGAGCTGAACAGG	NM_000478
	Reverse	GTCAATTCTGCCTCCTTCCA	
*opn*	Forward	TCAGCTGGATGACCAGAGTG	NM_001040060
	Reverse	TTGGGGTCTACAACCAGCAT	
*runx2*	Forward	TCTTAGAACAAATTCTGCCCTTT	NM_001024630.3
	Reverse	TGCTTTGGTCTTGAAATCACA	
*gapdh*	Forward	TGTTCGTCATGGGTGTGAAC	NM_002046
	Reverse	GTCTTCTGGGTGGCAGTGAT	

## Data Availability

Data sharing is not applicable to this article.
